# Supplementation of a new combination of prebiotic and postbiotic shapes fecal microbiota of old dogs while influencing immune parameters

**DOI:** 10.1038/s41598-025-10280-y

**Published:** 2025-08-04

**Authors:** A. Rodiles, W. Wambacq, C. Le Bourgot, E. Cox, F. Barbe, M. Hesta, E. Apper

**Affiliations:** 1 Lallemand Animal Nutrition, Blagnac, France; 2 Equine and Companion Animal Nutrition, Department of Morphology, Imaging, Orthopedics, Rehabilitation and Nutrition, Faculty of Veterinary Medicine, Ghent University, Salisburylaan 133, 9820 Merelbeke, Belgium; 3 Tereos, Moussy-le-Vieux, France; 4 Laboratory of Immunology, Department of Translational Physiology, Infectiology and Public Health, Faculty of Veterinary Medicine, Ghent University, Salisburylaan 133, 9820 Merelbeke, Belgium

**Keywords:** Immunosenescence, Elderly, Dog, Microbiota, Prebiotic, Postbiotic, Microbiome, Animal physiology

## Abstract

Healthy senior dogs were subjected for 77 days to a dietary regime with (or without) prebiotic + postbiotic mixture composed by short-chain fructo-oligosaccharides and yeast fractions (scFOS+). Their fecal microbiota was studied and the links between the microbiota and immune parameters were investigated after Lyme vaccination. All along the study results showed a clear modulation of the microbiota with a higher relative abundance (RA) for *Megamonas* spp., Bacteroidaceae, *Bacteroidetes plebeius*, Clostridiales, *Phascolarctobacterium*, Succinivibrionaceae, *Fusobacterium* spp. in feces from scFOS + dogs. An amplicon sequence variants (ASVs) index (Enterobacteriaceae + *Clostridium spiroforme* vs. *Fusobacterium* + *Megamonas*) was developed and found significantly different between the groups with scFOS + dogs showing a lower index suggesting a possible modulation of the physico-chemical environment in the gut, favoring the growth of strict anaerobes producing short-chain fatty acids. Phylogenetic Investigation of Communities by Reconstruction of Unobserved States (PICRUSt2) analysis revealed stimulation of propionate and acetate production pathways, vitamin biosynthesis (vitamin B2, B5 and B9 precursors) and pathways related to tricarboxylic acid cycle in the scFOS + group. The RA of several ASVs from Bacteroidetes and Fusobacteria families were found moderately negatively correlated to IgA and IgG concentrations (*P* < 0.05). In particular, *Megamonas* and *Phascolarctobacterium* appeared as interest genera. In summary, scFOS + is a good candidate to support the health of elderly dogs through microbiota changes. Metabolomics or in vitro mechanistic experiments will be crucial to further understand the mechanisms at play.

**Supplementary Information:**

The online version contains supplementary material available at 10.1038/s41598-025-10280-y.

## Background

As humans, elderly pets are subjected to immunosenescence^1^ and may present an altered immune response with a reduced ability to respond to new antigens and a constant presence of low-level inflammation, characterized by higher concentrations of pro-inflammatory cytokines. In recent years, the role of gut microbiota in modulating immunity has become an important research topic. Researchers have observed in rodents and humans that there is an association between gut microbiome changes and host age, although the precise mechanisms underlying the association of aging and gut microbiome are still unclear^2^. Xu *et al*.^3^ reported that in dogs gut microbiota structure differed according to their age and they observed that supplementation with probiotics may change this structure and the immune functions, especially in elderly dogs. One hypothesis is that the gut microbiota can influence several important physiological and metabolic functions of the host and drive immune homeostasis. A study performed by Thevaranjan *et al*.^4^ using young and old germ-free, and conventional mice revealed that mice under germ-free conditions are less prone to age-associated inflammation, exhibiting less pro-inflammatory cytokines in the blood than conventional mice. They also demonstrated that intestinal permeability increases with age due to age-related microbiota modifications. Microbial products that enter the blood stream trigger systemic inflammation ultimately altering macrophage function; illustrating that “aging” microbiota may promote inflammation. Thus, there is a strong interest to understand the interplay between gut microbiota and immunity to support health of elderly pets by mitigating adverse effects of immunosenescence and inflammation.

Diet is a key modulator that affects the gut microbiome. As the link between the hallmarks of immunosenescence and nutrition is increasingly studied, we are now entering into an era where animal health can be improved through dietary interventions. The primary focus of dietary strategies based on prebiotics is to enhance the immune fate of the intestinal mucosa by modulating the gut microbiome. We previously demonstrated in a study with old healthy dogs that a recently patented prebiotic and postbiotic mixture (Profeed ADVANCED^®^, from here scFOS+) was able to modulate several traits of immunosenescence, notably the serum ratio of CD4^+^:CD8^+^ T cells and the serum concentration of IgA^5^. No significant effect was found on the immune response after Lyme vaccination, or when comparing blood cytokine concentrations, potentially due to the vast intra-individual variability while using a limited number of dogs. The objective of the current article is to describe the effect of scFOS + on the fecal microbiota of the dogs from this previous study by Wambacq *et al.*^5^.

## Materials and methods

### Animals, diets, and experimental design

The analysis of the gut microbiota was performed from samples obtained in a previous study^5^. Written informed consent was obtained from the owners prior to their participation in the study. The research protocol was reviewed and approved by the Ethical Committee of the Faculty of Veterinary Medicine, Ghent University, Belgium (EC 2017/103) and was in accordance with institutional and national guidelines and regulations for the care and use of animals. All the methods reported were in accordance with the ARRIVE guidelines.

**Fig. 1:**
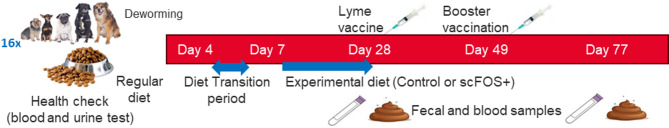
Experimental design of the trial, adapted from Wambacq *et al*. (2024). 16 dogs were included after passing a health screening (blood, urine and lyme antibody test) and other inclusion criteria, and were dewormed 3 days before day 0. From day 4 to day 7 dogs per group were adapted to the experimental diet (Ccontrol or scFOS+) for 3 weeks until lyme vaccination at day 28 (collecting fecal and blood samples right before), then booster vaccination at day 49. The final fecal and blood samples were collected at day 77.

In brief, 22 client-owned senior dogs (following Fortney scale^6^) were randomly assigned to one of two groups (scFOS + *versus* Control), taking gender, age, body weight and body condition score into account (Supplemented Table 1). The first group of dogs received an extruded dry diet for adult dogs (formulated following National Research Council requirements^7^ as stated in Wambacq *et al*.^5^, with 21.1 ± 0.8 of crude protein, 8.0 ± 0.9 crude lipid, 2.0 ± 0.4 crude fiber, 6.6 ± 0.6 ash, 62.4 ± 1.1 Nitrogen-Free Extract and 1.6 ± 0.02 metabolizable energy (mean ± SD of dry matter %)), hereinafter referred to as ‘Control diet’, and the second group received the exact same diet supplemented before extrusion with 1.1% Profeed ADVANCED^®^ (Beghin-Meiji, France), a compound feed combining scFOS prebiotic fibers with a new yeast postbiotic composed of various components from *Saccharomyces* and non-*Saccharomyces* strains (Beghin-Meiji; France), referred to as ‘scFOS+’ group for 14 consecutive weeks with a previous 7 days gradual transition to the new diet. Before the experiment started dogs were subjected to a complete health screening including a clinical examination, blood and urine analysis^5^, Lyme test was also performed (Canine Lyme Antibody Rapid Test, Abaxis Europe GmbH, Griesheim, Germany) and dogs were also dewormed (Caniquantel Plus, Fendigo sa/nv, Brussels, Belgium) 3 days before day 0. The inclusion criteria were: dogs should not be on any medication, they should be fed with dry food (kibbles) with body condition scores between 3 and 6 (0–9 score), have normal fasting blood outcomes and absence of protein in the urine, their core vaccination status should be completed from early life. Dogs accepted and tolerated very well the diets and there were no symptoms of gastrointestinal affection through the trial. The dogs were subjected to a first and a second Lyme vaccination at day 28 and 49, fecal and blood samples were collected at day 28 and day 77 (Fig. 1). Microbiota analyses were performed on a total of 16 dogs, due to five dogs could not finish the trial and few samples had low quality of DNA: 6 females and 2 males dogs for Control group, and 3 females and 5 males for scFOS + group.

**Table 1:** Summary of blood parameters, immune characteristics and fecal pH in old dogs fed with or without scFOS + for the average of the 2 time points (D28 and D77) used for the correlations with the fecal microbiota. Adapted table from Wambacq *et al*. (2024) where fecal microbiota analysis was concurrently performed.

Parameter	*N*	Control	*N*	scFOS+
Leucocytes, /µL	15	7275 ± 3443	14	6014 ± 1630
Total neutrophils, /µL	15	4848 ± 3045	14	4089 ± 1379
Total basophils, /µL	15	17.2 ± 12.2	12	15.2 ± 13.6
Total eosinophils, /µL	15	408 ± 236	13	297 ± 214
Total lymphocytes, /µL	15	1696 ± 658	14	1314 ± 506
Total monocytes, /µL	15	306 ± 133	14	297 ± 92
% CD4^+^CD8^−^T lymphocytes	15	41.3 ± 13.2	14	54.6 ± 15.7
% CD4^−^CD8^+^ T lymphocytes	15	41.5 ± 13.6	14	30.1 ± 13.2
CD4^+^: CD8^+^ ratio	15	1.24 ± 0.99	14	2.82 ± 2.85
Serum IgG, mg/dL	14	2005 ± 1073	13	1743 ± 793
Serum IgA, mg/dL	15	298 ± 137	13	178 ± 80
Serum IgM, mg/dL	15	2549 ± 1546	13	2382 ± 1221
Blood IL17, pg/mL	13	1040 ± 2134	9	107 ± 68
Blood TNF-alpha, pg/mL	7	70.5 ± 98.8	7	82.3 ± 93.0
Blood IL6, pg/mL	12	135 ± 127	7	123 ± 119
Nb IgA ASC per 500 000 PBMC	11	1938 ± 2379	11	776 ± 642
Borrelia specific IgA ASC per 500 000 PBMC	12	1.57 ± 2.02	8	3.21 ± 5.85
Area % of the well covered IgA Borrelia ASC	10	2.71 ± 3.09	8	2.51 ± 3.24
Borrelia specific IgG	11	168 ± 250	11	197 ± 298
Faecal pH	15	7.03 ± 0.36	14	6.98 ± 0.22

Data are mean ± SD. N: number of samples.

### DNA extraction and PCR of the fecal microbiota on V3−V4 region of the 16S rRNA genes

Fecal samples were collected by dog owners 24 hours before the consultation at D28 (prior to vaccination) and D77. The collection was done within 15 minutes after defecation and samples were promptly stored at −20 °C in sterile containers. The frozen samples were then transported to the Veterinary clinic of Ghent University in cooled condition and were subsequently stored at −80 °C until further processing. Previously to DNA extraction the 200 mg of samples were subjected to a lysis step (100 mM Tris pH 8, 100 mM Na EDTA pH 8, 100 mM NaCl, 1% (w/v) polyvinylpyrrolidone, 1% PVP40, and 2% (w/v) sodium dodecyl sulfate), with 0.3 g of zirconium beads (0.1 mm diameter) for 3 min at 2000 rpm in PowerLyzer (Qiagen, Venlo, the Netherlands), then DNA was extracted with phenol/chloroform extraction at the Center for Microbial Ecology and Technology (Ghent University, Belgium). DNA concentration and quality were verified with 260/280 and 260/230 ratios, using a DeNovix DS (Thermo Fisher Scientific, Waltham, MA, United States). PCR was done with universal primers 341F (5’-CCT ACG GGN GGC WGC AG -3’) and 785Rmod (5’-GAC TAC HVG GGT ATC TAA KCC-3’) according to Klindworth *et al*.^8^. PCR products were verified by gel electrophoresis, purified using the Promega Wizard PCR clean-up kit (Promega, Madison, WI, United States) following the manufacturer’s instructions, AMPure XP beads (Beckman-Coulter, Krefeld, Germany) and quantified with the QuantiFluor dsDNA System kit (Promega, Leiden, The Netherlands). High-throughput amplicon sequencing of the V3-V4 hypervariable region of the 16S rRNA genes were performed on a Illumina Miseq at LGC genomics GmbH (Berlin, Germany).

### Bioinformatics

Raw fastq files were imported, demultiplexed and processed using QIIME 2 (version 2020.2)^9^. Paired-ends fastq files were truncated by primer length, quality filtered and dereplicated through high resolution sample inference with DADA2^10^. MAFFT and FastTree2 were used for *de-novo* alignment and phylogeny construction^11,12^. Taxonomy was assigned to the resulting 16S rRNA marker genes against Greengenes (gg-13-8-99-nb-classifier, trained with naive-bayes for 341 F-785R region of the 16S) using sklearn classifier method to determine the taxonomy^13^. Low frequency amplicon sequence variants (ASVs; <100 reads in < 2 samples) were removed previously to taxonomical statistical analysis. Alpha and beta diversity were rarefied computed at 14,500 sequences depth and visualized by box plots (indexes) and non-metric multidimensional scaling (NMDS) ordination plots and data ellipses of significant groups at 0.75 SD using ggplot2^14^ and unweighted UniFrac distances. Compositional beta-diversity based on Aitchison distances^15^ was used to identify the discriminant ASVs with DEICODE^16^, then QURRO^17^ was used to test specific log ratios of discriminant ASVs based on Morton *et al.*^18^. The Phylogenetic Investigation of Communities by Reconstruction of Unobserved States (PICRUSt2) approach was used with default parameters to evaluate the functional potential of microbial communities^19^, obtaining the Kyoto Encyclopedia of Genes and Genomes (KEGG) abundance table and extracting the function with MetaCyc-Pathway-Classes database.

### Statistical analysis

 Differences obtained between alpha-diversity indexes were evaluated with non-parametric Kruskal-Wallis ranking test independently for each day and longitudinally from day 28 to day 77 and Beta-diversity with PERMANOVA. Taxonomical differential analysis was performed with ANOVA of the communities (ANCOM)^20^ at phyla level and with ALDEx2^21^ and longitudinal Kruskal-Wallis test^22^ at ASV level to evaluate the significance of the effect. All analysis were made with QIIME2. Spearman correlations were made in Python (version 3.7.6) to study relationships between immune parameters and alpha-diversity indexes of discriminating ASVs from ALDEx2 and DEICODE analysis. Results are represented by the mean value and standard deviation (SD) and standard error (SE) for PICRUSt2 representation. *P-value* was considered significantly different when *P* < 0.05, and trends when 0.05 < *P* < 0.1.

## Results

A total of 1,249,303 sequences of 373 ± 17 base pairs were obtained from 29 samples in high-throughput sequencing. After denoising and chimera filtering 744,329 sequences corresponding to 668 ASVs were kept for down-stream analysis. After a conservative filtration, 248 ASVs corresponding to 698,254 sequences were kept for the taxonomical analysis. Rarefactions curves showed a *plateau* in observed ASVs and Goods coverage close to 1 from 5000 sequences, reflecting that microbiota was fully sampled (supplementary Fig. 1). Table 1 summarizes the blood, immune and fecal pH values from each group used for microbiota analysis from Wambacq *et al.*^5^. Immune parameters followed normal physiological ranges with typical individual variability. Further experimental details and interpretation of immune parameters were reported elsewhere^5^.

### Fecal microbiota alpha-diversity

No treatment effect was observed on the different alpha-diversity parameters at D28. However, Shannon index and the number of observed ASVs significantly decreased 7 weeks after vaccination (D77) in dogs fed scFOS + when looking at transversal analysis on each day independently, but longitudinally paired Kruskal-Wallis analysis showed no differences between diets from day 28 to day 77, only a trend of Shannon and Evenness in the scFOS + group (*P* = 0.08) (Table 2).

**Table 2:** Alpha-diversity index at day 28 and day 77 and longitudinally from day 28 to day 77 in Control and scFOS + groups.

Treatment day	Shannon	Pielou_e	Faith_pd	Observed ASVs
Control D28 (*n* = 7)	4.7 ± 0.6	0.73 ± 0.07	7.2 ± 1.8	92 ± 28
scFOS^+^ D28 (*n* = 7)	4.6 ± 0.5	0.72 ± 0.04	7.3 ± 2.1	86 ± 22
*P-value*	0.338	0.180	0.949	0.898
Control D77 (*n* = 7)	4.5 ± 0.3^a^	0.70 ± 0.04	7.5 ± 2.5	91 ± 11^a^
scFOS^+^ D77 (*n* = 6)	↘3.9 ± 0.4^b^	↘0.64 ± 0.05	5.6 ± 1.9	71 ± 13^b^
*P-value*	0.032	0.116	0.116	0.037
*P-value D28-D77*	0.296	0.296	0.210	0.143

Superscripts per time point refers to significant differences between treatments (*P* < 0.05). Oblique arrows represent a trend on time effect within treatment (0.05 < *P* < 0.1). Data are mean ± SD.

**Table 3:** Relative abundance (%) of amplicon sequence variants (ASVs) that significantly changed or tended to change in the fecal microbiota of dogs supplemented or not with scFOS + according to the sampling time.

ASVs	Control D28	scFOS + D28	*P*-value
Enterobacteriaceae ASV	6.22 ± 15.1	0.33 ± 0.10	**0.09**
*Megamonas* spp.	0.95 ± 0.90	5.70 ± 7.90	0.10

	Control D77	scFOS + D77	*P-value*
Bacteroidaceae ASV	0.01 ± 0.02 ^b^	0.57 ± 0.47 ^a^	0.02
*Bacteroides* spp.	0.29 ± 0.29 ^a^	0.00 ± 0.00 ^b^	0.05
*Bacteroides* spp.	0.19 ± 0.20 ^b^	1.57 ± 2.23 ^a^	0.04
*Bacteroides* spp.	0.16 ± 0.37	0.49 ± 0.46	0.10
*Bacteroides plebeius*	0.09 ± 0.22 ^b^	0.69 ± 0.86 ^a^	0.03
Clostridiales ASV	0.20 ± 0.49	0.30 ± 0.38	0.06
Lachnospiraceae ASV	0.02 ± 0.05 ^b^	0.09 ± 0.08 ^a^	0.03
*Megamonas* spp.	3.26 ± 7.34 ^b^	16.4 ± 17.4 ^a^	0.05
*Phascolarctobacterium* spp.	0.02 ± 0.05	0.81 ± 0.85	0.07
*Fusobacterium* spp.	0.59 ± 0.91 ^b^	2.17 ± 2.37 ^a^	0.05
Succinivibrionaceae ASV	0.04 ± 0.11	0.57 ± 1.13	0.06

Superscripts refers to significant differences between treatments (*P* < 0.05). Data are mean ± SD.

### Microbial composition: taxonomy

The fecal microbiota was composed of 5 phyla: Firmicutes (RA of 67.8 ± 20.24%), Fusobacteria [RA of 12.8 ± 14.0%), Bacteroidetes (RA of 11.1 ± 10.2%), Actinobacteria (RA of 3.8 ± 4.7%), and Proteobacteria (RA of 4.5 ± 6.7%); all these phyla being observed in all dogs but with high inter individual variability. At family level, Lachnospiraceae (21.0 ± 17.4%), Fusobacteriaceae (12.5 ± 13.7%), Clostridiaceae (16.0 ± 12.5%), Erysipelotrichaceae (8.8 ± 12.6%), Veillonellaceae (8.1 ± 13.0%), Streptococcaceae (5.4 ± 16.8%), Bacteroidaceae (5.1 ± 6.9%) contributed to more than 75% of the RA whatever the sampling time and treatment, in lesser extent (RA < 5% on average) Lactobacillaceae, Paraprevotellaceae and Enterobacteriaceae. 21 ASVs detected with high frequency in our samples represented almost 60% of the microbiota. Among them, *Clostridium hiranonis* (14.7 ± 12.1% RA; 28/29 dog frequency), unidentified genus from Fusobacteriaceae (9.28 ± 9.60%; 28/29), *Megamonas* spp. (6.28 ± 11.7%; 23/29), *Blautia producta* (4.43 ± 4.07%; 29/29), *Bacteroides* (3.95 ± 5.94%; 24/29), *Ruminococcus gnavus* (3.69 ± 4.65%; 29/29), *Dorea* spp. (3.46 ± 3.78%; 28/29), unidentified genus from Lachnospiraceae (3.18 ± 5.29; 28/29), *Blautia* spp. (2.96 ± 4.19%; 28/29), *Fusobacterium* spp. (2.29 ± 3.87%; 24/29), *Blautia* spp. (1.86 ± 2.15%; 26/29), *Collinsella stercoris* (1.76 ± 1.94%; 27/29) and *Faecalibacterium prausnitzii* (1.54 ± 2.15%; 22/29).

### Microbial composition: differential analysis

**Fig. 2:**
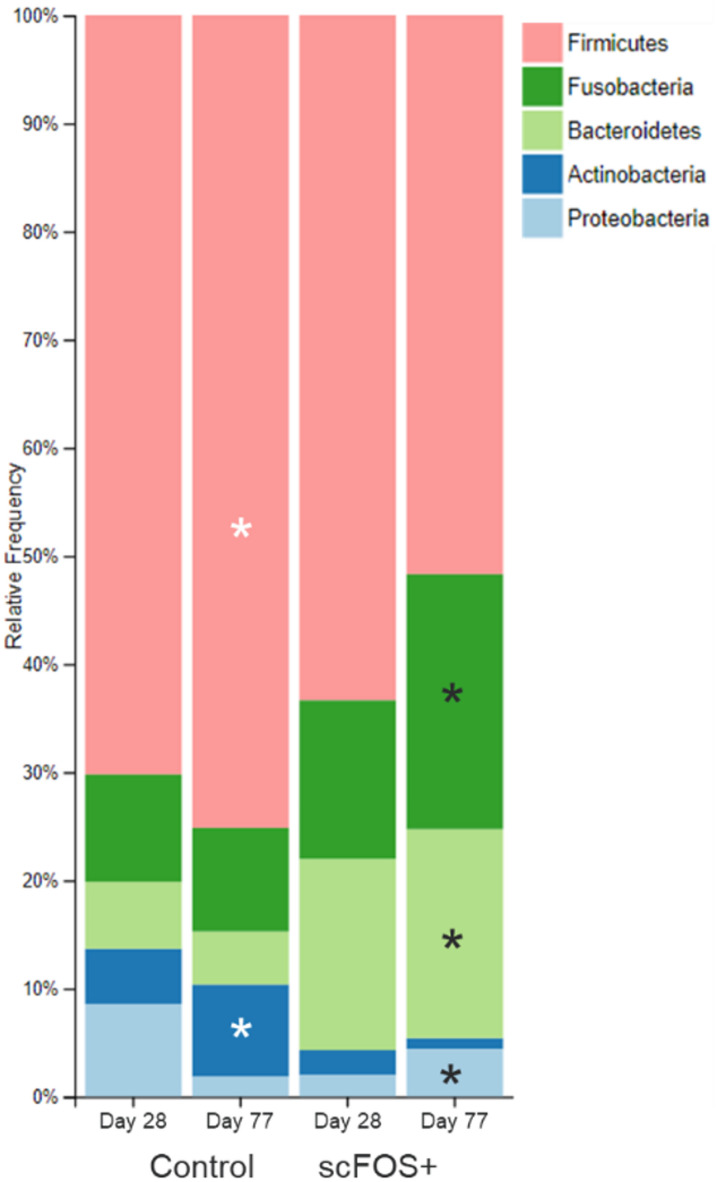
Relative abundance (%) of the phylum in Control and scFOS + at D28 and D77. Asterisk (*) represents significant higher relative abundance at phyla level at D77 between treatments using ANCOM analysis (*P* < 0.05).

Remarkably, significant differences (*P* < 0.05) at phyla level were found between treatments at day 77 with higher RA of Fusobacteria (10.4 ± 13.3% vs. 18.7 ± 17.4%), Bacteroidetes (7.5 ± 6.35% vs. 18.3 ± 9.8%), and Proteobacteria (1.79 ± 2.36% vs. 6.08 ± 6.25%) and lower abundance of Firmicutes (72.7 ± 16.7% vs. 55.5 ± 16.6%) and Actinobacteria (7.47 ± 9.98% vs. 1.26 ± 1.45%) in the scFOS + group vs. Control (*P* < 0.05; Fig. 2). ASVs related to Bacteroidaceae, *Bacteroides*, *Bacteroides plebeius*, *Fusobacterium*, Lachnospiraceae and especially *Megamonas* were significantly impacted, as well as other ASVs trend for a higher RA of ASVs related to *Phascolarctobacterium*, Succinivibrionaceae and unidentified Clostridiales (Table 3). When looking at data independently of time, most of the effects detected at D77 were significant, in addition to a trend for a higher RA of *Prevotella copri P* < 0.10; Supplementary Table 2). At day 28 only few trends could be observed (*P* < 0.10), a decrease in the RA of Enterobacteriaceae ASV and an increase in the relative abundance of *Megamonas* ASV in scFOS + group. Longitudinal comparison from day 28 to day 77 showed some trends between treatments in *Turicibacter* (*P* = 0.087) from 2.41 ± 3.69 to 0.42 ± 0.8% RA for scFOS+ (*P* = 0.068) where Control did not change (from 0.29 ± 0.46 to 0.32 ± 0.43% RA; *P* = 0.465) and in Enterobacteriaceae (*P* = 0.066) from 6.22 ± 14.15 to 0.07 ± 0.15% RA for Control (*P* = 0.080) where in scFOS + did not change (from 0.33 ± 0.85 to 3.44 ± 7.19% RA; *P* = 0.345). These changes were basically because of 2 dogs: a giant Lerse Wolfshond dog for decreasing *Turicibacter* (3 to 0.3%RA) while increasing Enterobacteriaceae (3 to 19% RA) in scFOS + and a large Witte herder dog for decreasing notably the Enterobacteriaceae from day 28 to 77 (44 to 0.5% RA) in Control.

### Microbial composition: beta-diversity

**Fig. 3:**
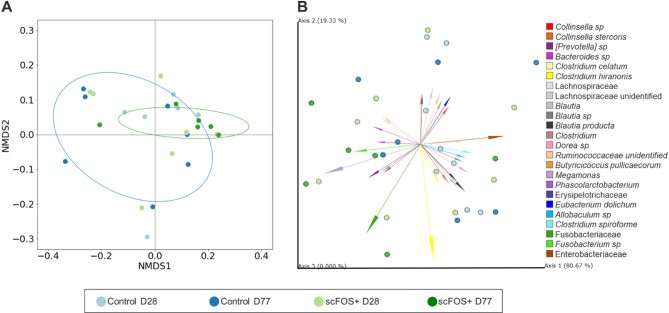
Non-metric multidimensional scaling (NMDS) of unweighted Unifrac distances betweenCcontrol and scFOS + during the experiment. Ellipses show 1.5 SD at D77 (*P* < 0.05) (**A**). Principal component analysis (PCA) of Aitchison distances of the treatments per time point with weight of the discriminant ASVs represented by arrows length from DEICODE. Data points represent individual samples and vectors are taxa driving differences in ordination space (**B**).

**Fig. 4:**
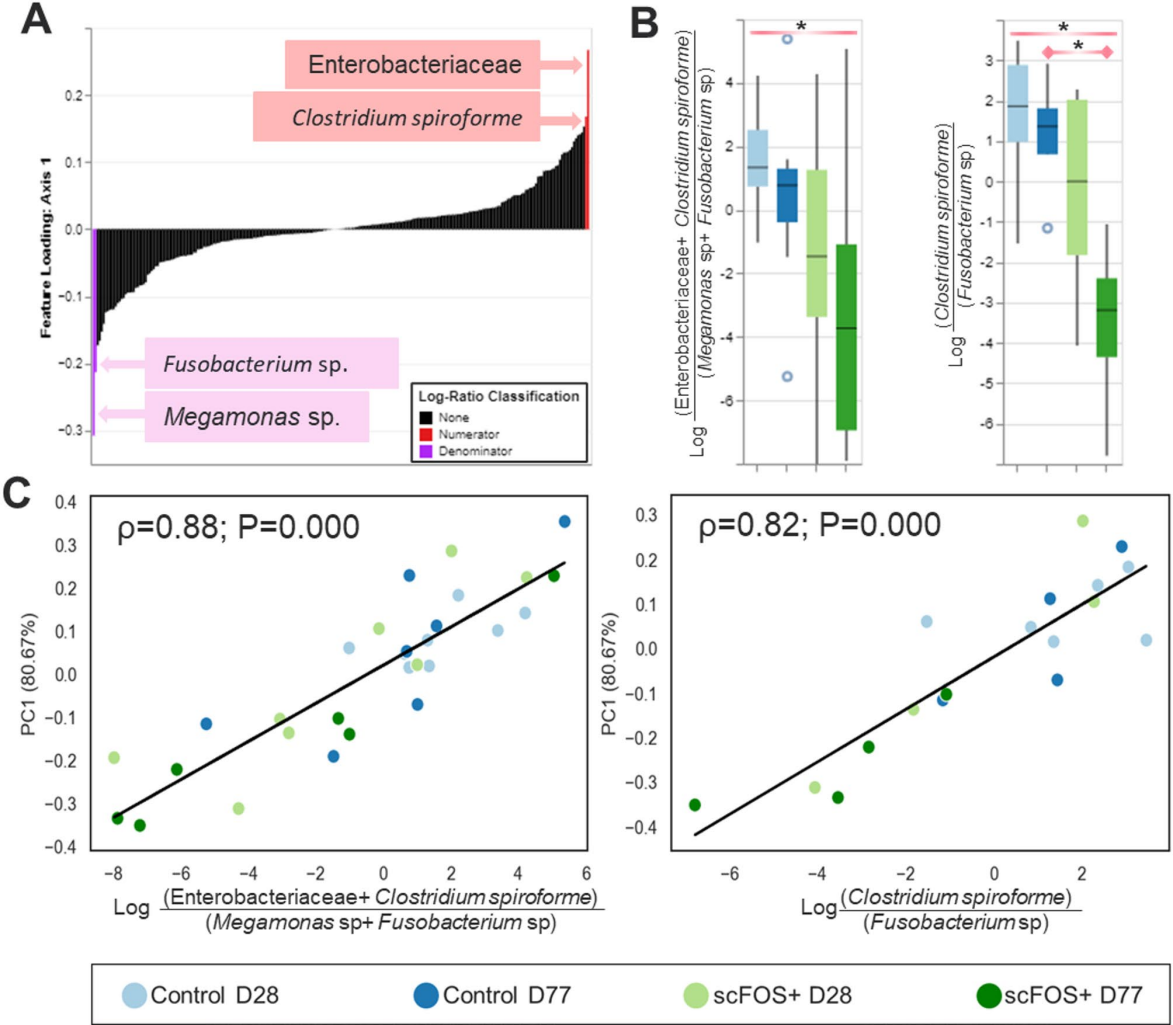
Ratio of taxonomic differences between Control and scFOS+. Ranking of principal component analysis (PCA) Axis 1 taxonomic loadings highlighting the 2 highest features loading amplicon sequence variants (ASVs) (**A**). Box plots of natural log ratios (Enterobacteriaceae + *C*. *spiroforme*)/(*Megamonas* spp.+*Fusobacterium* spp.) and (*C. spiroforme*/*Fusobacterium* spp.) per treatment time points (**B**) and significant correlated with PCA Axis 1 (ρ: Spearman correlation coefficient) (**C**). Horizontal bar with * shows significant differences between treatments (including both D28 and D77) and at D77 (*P* < 0.05).

At day 77 the composition of the communities significantly differed between treatments in unweighted distances (Fig. 3A). On the other hand, Aitchinson PCA analysis was used to obtain the discriminant ASVs to differentiate significantly the treatments with both Axis 1 (80.7% variability explained) and Axis 2 (19.3% variability explained) (Fig. 3B). Axis 1 was associated with more *Fusobacterium* spp., *Megamonas* spp., *Phascolarctobacterium* spp., *Bacteroides* spp., *Prevotella* spp. (Paraprevotellaceae family) and less Enterobacteriaceae and *C. spiroforme*. Opposite to Axis 2 that was characterized by an opposition between *C. hiranonis* and the families of Lachnospiraceae, Erysipelotrichaceae, Ruminococcaceae, and *Eubacterium dolichum.* We assessed the ratio between discriminant ASVs according to the treatment and the time point (Fig. 4). A significant lower ASV ratios of the log (Enterobacteriaceae + *C. spiroforme*)/(*Megamonas* spp. + *Fusobacterium* spp.) was found with the scFOS + supplementation whatever the time point (*P* < 0.05) and trends were observed at both time points (*P* < 0.10) (Fig. 4B). Similarly, the log (*C. spiroforme/Fusobacterium* spp.) was significantly lower with scFOS + all along the study (*P* < 0.01), at D77 (*P* < 0.05) and tended to be different at D28 (*P* < 0.10). The same observation was obtained for the log (Enterobacteriaceae*/Megamonas* spp.) at day 28 (data not shown). All those ratios were also significantly correlated with the first axis of the Aitchinson PCA, corroborating that only with the Axis 1 the differences in the treatments were explained (*P* < 0.0001; Fig. 4C), thus clearly showing 2 different microbiota profiles.

### Correlations between immune parameters and alpha-diversity index

We found moderate correlations (Spearman correlations between 0.30 and 0.45) between immune parameters and alpha-diversity index. More precisely, the CD4+:CD8 + ratio tended to be negatively correlated with Shannon index (trend, ρ =  −0.34; *P* < 0.10), Faith’s phylogenetic diversity (faith_PD; ρ = −0.42; *P* < 0.05) and evenness (Pielou; ρ =  −0.37; *P* < 0.05).

### Correlations between immune parameters and relative abundance of discriminant ASVs

Overall, the correlations between serum Ig and ASVs were moderate (0.30–0.60) to high (> 0.60), depending on the immune parameter considered.

#### Cytokines and ASVs

 Negative correlations were found between IL6 and Lachnospiraceae (ρ = −0.592; *P* < 0.01), *Blautia* spp. (ρ = −0.561; *P* < 0.05), *B. producta* (ρ = −0.529; *P* < 0.05), *Dorea* (ρ = −0.465; *P* < 0.05) and *Bacteroides* (ρ =  −0.426; *P* < 0.05).

#### Serum Ig and ASVs

 Total serum IgG concentration was negatively correlated to *Fusobacterium* spp. (ρ =   −0.421; *P* < 0.05), Bacteroidaceae (ρ = −0.487; *P* < 0.01), *Bacteroides* spp. (ρ =   −0.485; *P* < 0.01), *Prevotella* spp. (ρ =   −0.644; *P* < 0.01), while being positively associated with the log (*C. spiroforme/Fusobacterium* spp.) ratio (ρ =  0.606; *P* < 0.01). Serum *Borrelia* specific IgG was negatively associated with the *Turicibacter* (ρ =  −0.676; *P* < 0.01). Total serum IgA was negatively correlated with Proteobacteria (ρ =  −0.432; *P* < 0.05) and IgM positively correlated with Proteobacteria (ρ =   0.422; *P* < 0.05).

#### ELISPOT and ASVs

 Total number of IgA ASC in serum was negatively associated with the *Prevotella* (ρ = −0.536; *P* < 0.05), *Megamonas* (ρ = −0.462; *P* < 0.05), Veillonellaceae family (ρ =  −0.470; *P* < 0.05). When looking at the specific *Borrelia* IgA ASC, the % of covert area was positively correlated to Bacteroidetes (ρ = 0.591; *P* < 0.01), Bacteroidaceae (ρ = 0.640; *P* < 0.01), *Bacteroides* (ρ =  0.706; *P* < 0.01), *Prevotella* spp. (ρ =  0.713; *P* < 0.01), while the log (Enterobacteriaceae*/Megamonas*) ratio was negatively correlated (ρ =  −0.627; *P* < 0.05). In addition, the ratio (*Borrelia* specific IgA ASC/ total number of IgA ASC) was negatively correlated with the log (Enterobacteriaceae*/Megamonas)* ratio (ρ =  −0.975; *P* < 0.01), *Blautia* spp. (ρ =  −0.706; *P* < 0.01) and *B. producta* (ρ = −0.633; *P* < 0.05) and positively associated with Veillonellaceae (ρ = 0.615; *P* < 0.05), *Phascolarctobacterium* (ρ = 0.686; *P* < 0.05) and *Fusobacterium* (ρ = 0.640; *P* < 0.05).

#### Fecal pH and ASVs

 Fecal pH was positively correlated with Enterobacteriaceae (ρ = 0.487; *P* < 0.05) and negatively correlated with *Phascolarctobacterium* (ρ = −0.457; *P* < 0.05).

### Microbial function: PICRUSt2

**Fig. 5:**
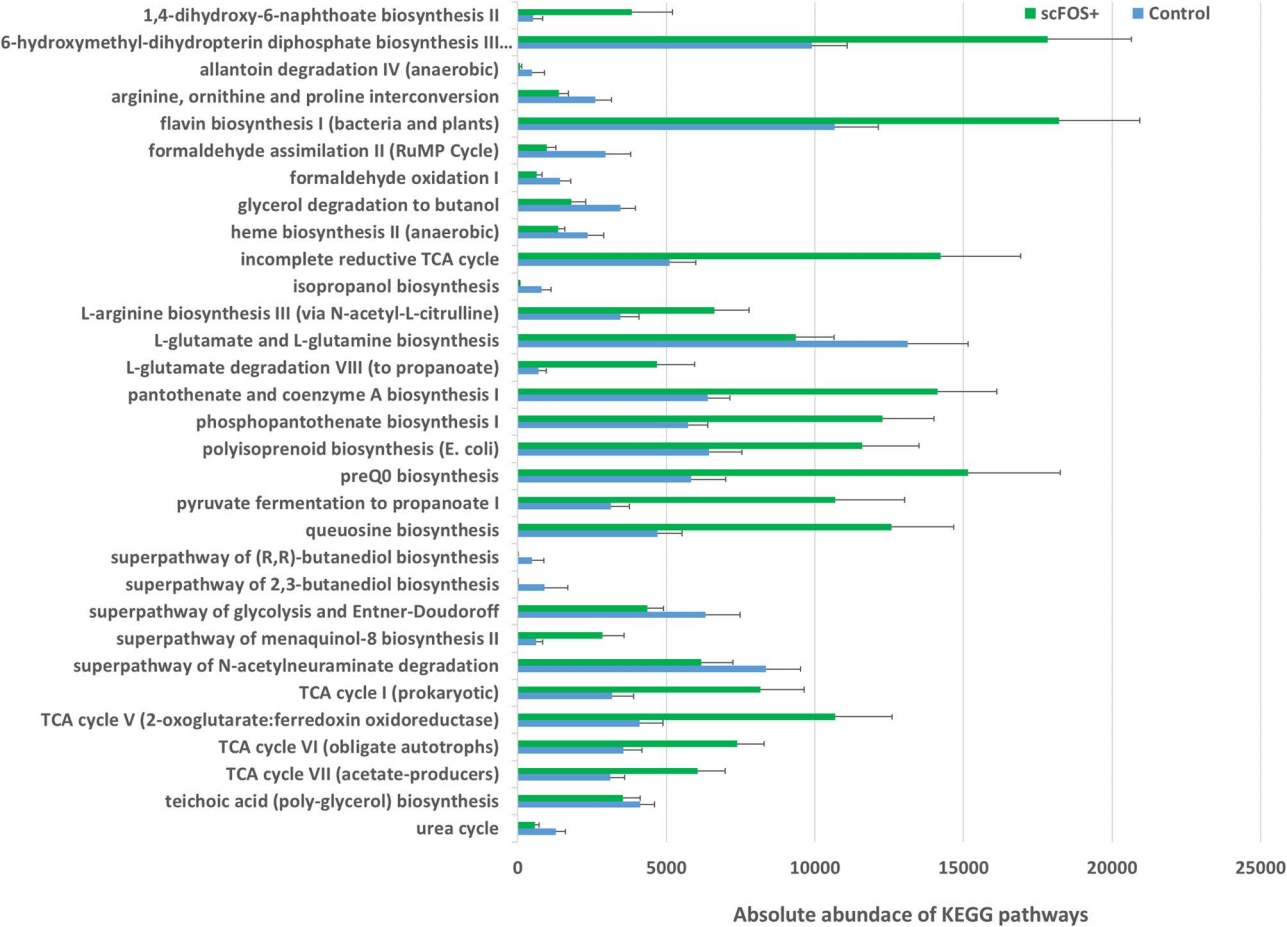
Absolute abundance of significant different MetaCyc pathway classes found between treatments (including both D28 and D77) with PICRUSt2 (*P* < 0.05) using ALDEx2 analysis. Results are mean ± standard error.

A total of inferential 306 distinct pathways were identified, with 32 showing significant differences between the microbiota of the Control and scFOS + groups (refer to Fig. 5). Among the 18 pathways upregulated with scFOS+, 4 were associated with vitamin B biosynthesis, 2 with vitamin K biosynthesis, 4 with the tricarboxylic acid cycle (TCA), 2 with propionate biosynthesis, and 1 with acetate biosynthesis. Conversely, among the 14 pathways overexpressed in the Control diet, 4 were involved in alcohol biosynthesis and 2 in formaldehyde metabolism.

## Discussion

The addition of prebiotics and postbiotics in the diet of elderly pets is proposed as an interesting nutritional strategy to manage chronic inflammation and mitigate negative effects of immunosenescence^23,24^. However, to our knowledge, little is known on their effects on the gut microbiota of elderly dogs, in relation to their immune parameters. We thus studied the impact of an original combination of a prebiotic and postbiotic (Profeed ADVANCED^®^) on the fecal microbiota and the link with previously reported effects on immune parameters of healthy elderly dogs. We hypothesized that scFOS + could improve some hallmarks of immunosenescence through modulation of the gut microbiota and/or through direct immune-stimulation.

### Our study allowed characterizing the gut microbiota of old dogs and shaping 2 different profiles

The fecal microbiota of the dogs was composed of five main phyla across the treatments. The phylum of Firmicutes emerged as the most dominant phylum, comprising 68% of relative abundance, followed by Fusobacteria at 13%, Bacteroidetes at 11%, and approximately 4% for Proteobacteria and Actinobacteria. This is consistent with recent studies involving dogs of varying ages^25–29^. The predominant families identified were Lachnospiraceae, Fusobacteriaceae, Clostridiaceae, Erysipelotrichaceae, Veillonellaceae, Streptococcaceae, Bacteroidaceae, Lactobacillaceae, Paraprevotellaceae, and Enterobacteriaceae. Notably ASVs associated with *C. hiranonis*, Fusobacteriaceae, *Megamonas*,* Allobaculum*,* Bacteroides* spp., *Blautia* (*B. producta*) and Lachnospiraceae accounted for over 50% of the total relative abundance and were prevalent among nearly all dogs. Despite the lack of extensive investigation into the microbiota of aging dogs to date^30,31^, a recent study involving 29 healthy companion dogs ranging from 3 to 13 years of age and representing various breeds^28^ reported a core microbiota consistent with our findings in elderly dogs.

We used a new approach, Aitchinson distances, in order to investigate the influence of ASVs on composition and interrelationship of fecal microbiota. Our findings suggest that *Fusobacterium* spp.*/*Fusobacteriaceae, *C. hiranonis*, Lachnospiraceae, Erysipelotrichaceae, *Blautia* spp., *Prevotella* spp., *Megamonas* spp., *Phascolarctobacterium* spp. and Enterobacteriaceae are key contributors. Remarkably, our data revealed distinct bacterial profiles. Notably, *C. hiranonis* was an important contributor that was negatively associated with Erysipelotrichaceae, Lachnospiraceae and Ruminococcaceae and *E. dolichum* ASVs, while being positively associated with *Blautia* spp., *B. producta* and Fusobacteriaceae. Interestingly, these opposed bacteria may compete for the same substrates and share overlapping functions, like short-chain fatty acid (SCFA) production from carbohydrates or proteins. Furthermore, in humans *E. dolichum* has been linked to frailty^32^, while in dogs, *C. hiranonis*,* Blautia* spp., and *Fusobacteriaceae* are considered as key bacteria to assess microbial changes in subjects suffering from chronic inflammatory enteropathy^33^. In addition, *C. hiranonis* is associated with normal bile acid metabolism^34^. Bile acid metabolism is an important pathway not just for lipid digestion, but also for regulating intestinal inflammation, which is commonly altered in chronic gastrointestinal diseases, making this bacterium an interesting biomarker and a potential new probiotic candidate for dogs. Importantly, some bacteria belonging to the Erysipelotrichaceae family have also been described as key microbes in bile acid metabolism^35,36^. Those observations suggest that we shaped two profiles of microbiota, potentially competing for the same substrates and involved in similar functions, maybe depending on the phenotype/genetics/metabolism of the host. More research is needed to better understand the biological significance of these microbiota profiles on the host, as well as the impact of the host characteristics on those profiles.

### The combination of prebiotic and postbiotic modulated the gut microbiota of aged dogs

ScFOS + diet led to microbial composition modulation, especially after 77 days of supplementation and following vaccination. At this time point we observed higher RA of Fusobacteria, Bacteroidetes and Proteobacteria and lower RA of Actinobacteria and Firmicutes phyla. Still, it remains difficult to interpret these results which could be partially attributed to the lower alpha-diversity. The proportion of Firmicutes respect to Bacteroidetes decrease with scFOS at both Day 28 and 77. The Firmicutes/Bacteroidetes ratio is considered as a potential biomarker of health in humans, this ratio has been reported to increase in humans fed with high levels of fibre^37^, tend to increase with age^38^, and be higher in obese and type 2 diabetes mice fed with live yeast^39^. In puppies Bacteroidetes was found to be associated with puppies weight at birth, being minimum in low birth weight puppies^26^. In addition, a decrease in fecal Bacteroidetes from 11 to 9% has been observed in dogs with chronic enteropathy^40^. We also reported a significant decrease in RA of Actinobacteria and an increase of Fusobacteria in ScFOS+. Interestingly a recent study performed to compare the microbiota of young adult vs. old dogs reported that despite the fact the main phyla remained unaffected, an increase in RA of Actinobacteria was associated with memory failures, and the RA of Fusobacteria phylum was negatively associated with age^28^.

At a deeper taxonomic level, all along the study and particularly at day 77, *Megamonas* spp., Bacteroidaceae, *B. plebeius*, Clostridiales, *Phascolarctobacterium*, Succinivibrionaceae, *Fusobacterium* spp. were found in higher RA in feces from scFOS + dogs. Most of these taxa are present in high abundance in fecal microbiota of healthy adult dogs from 0 to 10 years old^26,27,41^. In humans, some authors associated *Bacteroides* with celiac or IBD disease, however, the association must be done at a species level (and even at strain level)^42^ rather than at genus level. Indeed, infants with high genetic risk to develop celiac disease had a reduced prevalence of *B. ovatus*,* B. plebeius*, and *B. uniformis*, and an increased prevalence of *B. vulgatus* compared with infants with low genetic risk^43^, underlining the necessity to associate the microbiota evolution with metabolic/phenotypic traits of the microbes or the host. Interestingly, the genomes of Bacteroidetes contain polysaccharide utilization *loci* that encode the tools required to utilize complex carbohydrates, and certain *Bacteroides* encode proteins predicted to display α-mannosidase or α-mannanase activity, making them able to metabolize the major α-mannose-containing glycans from different yeast species^44^. This could explain why in our study Bacteroidetes harboured higher relative abundance in microbiota from scFOS + fed dogs.

As the beta-diversity significantly changed with scFOS+, we identified key bacteria that could explain change in the community structure. We discriminated the fecal microbiota structure from dogs fed scFOS + and Control diets by identifying which bacteria contributed to the discrimination. *Fusobacterium* spp. and *Megamonas* spp. on the one side (denominator), Enterobacteriaceae and *C. spiroforme* on the other side (numerator) significantly opposed the scFOS + and the Control groups, respectively. Genera *Fusobacterium* and *Megamonas* were associated with *Phascolarctobacterium*, Fusobacteriaceae and *Prevotella* spp. In addition, even if scFOS + did not affect the relative abundance of *F. prausnitzii* (1.2% and 1.9% for Control and scFOS+, respectively), the species was found to be associated with Fusobacteriaceae (data not shown). Unlikely to what is observed in humans, *Fusobacterium* in dogs is associated to a healthy microbiota^31^ and can produce SCFA from different amino acids. The genus *Phascolarctobacterium* spp. produces high amounts of acetate and propionate and can use succinate produced by other bacteria^45,46^. It is interesting to underline that this genus was associated with *B. plebeius* and Succinivibrionaceae, which are potential succinate producers. Wu *et al*.^46^) found a decrease in relative abundance of *Phascolarctobacterium* in elderly humans; while this genus has been associated with a positive mood^47^. Interestingly, a decrease in several species of *Megamonas*,* P. copri* and *B. plebeius* has been observed in dogs suffering from canine anal furunculosis^48^. *Megamonas* is an important propionate and acetate producer and is a common gut commensal microbe of carnivorous animals. Increased abundance of *Megamonas* have been found in healthy compared to diarrheic cats^49^ and in dogs consuming inulin-rich diets^50^. Based on these results, a greater emphasis on Veillonellaceae family and its potential impact on dog gastrointestinal health may be justified in the future.

The significant opposition obtained between the (Enterobacteriaceae *+ C. spiroforme)* and *(Megamonas* + *Fusobacterium)* and the crosstalk existing between the different ASV to produce SCFAs, support the hypothesis that scFOS + could modulate the physico-chemical environment in the gut, leading to better conditions for the growth of strict anaerobes producing SCFAs, even after vaccination. The family of Enterobacteriaceae is composed of facultative anaerobe bacteria so that they take advantage of the oxygen available in the intestine. In addition, they efficiently use various substrates among which sugars and proteins. In agreement with our hypothesis, we obtained a positive correlation between the relative abundance of Enterobacteriaceae and fecal pH while being negatively correlated with *Phascolarctobacterium*. Taken all together, our results suggest that scFOS + resulted in stabilizing microbiota by favouring SCFAs producing bacteria using different substrates and cross-feeding. Although we did not measure SCFAs concentration, propionate is primarily produced by Bacteroidetes and some Firmicutes, among which *Phascolarctobacterium*^51^ and *Megamonas* spp. Of note, PICRUSt2 analysis revealed the stimulation of propionate and acetate production pathways in the scFOS + group.

Beside the activation of some acetate and propionate production pathways, functional inference revealed that adding scFOS + resulted in increased RA of KEGG pathways related to B vitamin biosynthesis, especially B2 (riboflavin), B9 precursor (pterin) and B5 and different pathways related to the TCA cycle. In healthy adult dogs on complete and balanced diets, vitamin B deficiencies are not common as commercial diets contain enough B vitamins. However, in case of chronic gastrointestinal disease (e.g. pancreatic insufficiency), dysbiosis or anemia, vit B12 and folic acid deficiency are common which could be the case of Senior dogs^52^. Bacterial vitamin B2 exists as free riboflavin and is directly absorbed in the large intestine. Vitamin B2 levels are important for T cell differentiation^53^. A vitamin B2 deficiency suppresses the activity of acylCoA dehydrogenases, involved in the oxidation of fatty acids to generate acetyl-CoA^54^; while B5 vitamin, pantothenate and phosphopantothenate are precursors of acetyl CoA^55^. Fatty acid oxidation is involved in the activation, differentiation and proliferation of immune cells through the generation of acetyl CoA and its entry into the tricarboxylic acid cycle^54^. In addition, the same authors reported how the balance between B2 and B9 vitamins was key to understand immune homeostasis. The commensal bacteria are both providers and consumers of B vitamins. Interestingly, *P. copri*,* F. varium* express factors essential for vitamin B2 and B9 synthesis, suggesting that these bacteria are important sources of microbial vitamin B2 and B9 in the large intestine. In addition, recent evidence indicates that some species in Bacteroidetes phylum produce more riboflavin (B2 vitamin) than Firmicutes or Actinobacteria^56^. Those observations are consistent with our findings. However, given the limitations of PICRUSt2 as solely a predictor of metagenomic function, a true experimental validation through is necessary to confirm these predicted functions.

### How scFOS + could play a role to shape immune parameters through a gut microbiota modulation?

 The last part of our work was dedicated to the analysis of some statistical correlations between different immune parameters and ASVs. Total serum IgM concentration was positively, while total serum IgA was negatively, correlated with higher Proteobacteria RA. The number of IgA secreting cells was negatively associated with Veillonellaceae family, *Megamonas* spp. and *Prevotella* spp. Finally, total serum IgG concentration was negatively associated with RA of Bacteroidaceae, *Bacteroides*,* Prevotella* and *Fusobacterium* while positively associated with the ratio between *C. spiroforme* and *Fusobacterium*. Overall, the microbiota as shaped with the scFOS + supplementation was correlated to less concentrations of total serum IgG and IgA, the latter being found significantly lower^5^. Total serum IgA and IgG contents increased with age and chronic inflammatory disorders in humans^57,58^, but also with age in dogs^59^. Thus, we could speculate that the supplementation with scFOS + allowed decreasing the pro-inflammatory status, in agreement with the numerically lower concentrations of IL6 (an Ig synthesis inducer) and IL17, and the significant increase in CD4^+^:CD8^+^ ratio^5^.

The specific immune response against *B. burgdoferi* vaccine was also investigated. In this case, serum *Borrelia* specific IgG concentration was negatively correlated with the genus *Turicibacter*, a genus formerly described as a butyrate-producer, decreasing with hemorrhagic diarrhea in dogs^60^ and in dogs suffering from chronic enteropathy^33^, as it seems the case of the giant dog fed with scFOS + in the present experiment. Interestingly, the *Borrelia* specific IgA covert area was positively correlated with Bacteroidetes, Bacteroidaceae, *Bacteroides*,* Fusobacterium*,* Prevotella* and negatively (ρ=-0.975) with the ratio between Enterobacteriaceae and *Megamonas*. We can hypothesize that the production of specific *Borrelia* IgA, but not the proliferation of the cells, was stimulated by the ASVs that were significantly modulated with scFOS+, even if the biological causation remains unclear. No correlation was found between the number of specific *Borrelia* IgA secreting cells and ASVs while the ratio between the number of IgA secreting cells over the number of *Borrelia* specific IgA secreting cells was positively associated with Veillonellaceae, *Phascolarctobacterium* and *Fusobacterium*; however being negatively associated with *Blautia* and *B. producta*. Those results confirm the potential interest of deeper investigating the functional role of *Megamonas* spp. and *Phascolarctobacterium* spp. not only for gut health indices, but also for more systemic health parameters in the canine species.

It is worth to note that this work is preliminary, and the interpretation should be taken cautiously. First, even if we applied strict exclusion and inclusion criteria, our canine cohort was small, resulting in a small sample size (due to the difficulty to recruit and follow a high number of elderly dogs) for statistical analysis. Second, the study was focused around Lyme disease vaccination, however the study lacks on microbiota baseline data at day 0. Thirdly, due to our limited sample size, the correlations we performed were Spearman correlations not adjusted by the multi-comparisons, so that we cannot exclude the presence of false-positives. Lastly, several of those correlations – but not all – were considered as moderate, thus comprised between 0.30 and 0.60. We therefore need to go further to deepen our conclusions, notably by increasing the sample size, and, ultimately, by going from a statistical relationship to a causation relationship, by, for example, designing specific *in vitro* models to decipher possible links between some ASVs and inflammation or even Ig induction effect. However, such kind of data obtained in elderly owned dogs is still very scarce, and we think that this work, although imperfect, paves the way for a better understanding between the microbiota, the inflammation and the immune response of elderly dogs.

## Conclusion

This study showed how a new ingredient composed of prebiotic combined with specific yeast fractions (Profeed ADVANCED^®^) can shape the gut microbiota composition and community structure of elderly dogs and modulate the CD4^+^:CD8^+^ ratio and total serum IgA, known to decrease and increase with age respectively. To our knowledge, this is the first time that such a change in microbiota has been demonstrated in old dogs. There are some limitations to our study that could potentially impact on the interpretation of the findings among which the low number of dogs, absent of fecal microbiota day 0 and the lack of “sentinel” animals. Further research is required to better understand the impact on specific immune responses, but also on the functional impact of the change in microbiota. Notably, we could imagine going further immune cell phenotyping, to be able to see what type of cells have been changed with the prebiotic and postbiotic mixture, as well as metabolomics in both blood and feces. Also, validating the putative role of bacteria on immunity would deserve more attention by performing classical microbiology and *in vitro* mechanistic experiments. Having a complete picture of what happens in the holobiont will be crucial to further optimize the use of nutritional strategies to support elderly dogs.

## Electronic supplementary material

Below is the link to the electronic supplementary material.

Supplementary Material 1

## Data Availability

Sequence data that support the findings of this study have been deposited in the NCBI with the primary accession code PRJNA1183820.
