# Supplementary material for: Supplementation of a new combination of prebiotic and postbiotic shapes fecal microbiota of old dogs while influencing immune parameters
Source: Sci Rep. 2025 Aug 4;15:28447. doi: 10.1038/s41598-025-10280-y (PMC12322041; doi:10.1038/s41598-025-10280-y)

**Supplementary information**

**Supplementary Table 1**. Breed, age, Gender, Body Condition Score (BCS), initial and final Body weight (IBW, FBW, respectively), Senior state and diet of dogs included in the study adapted from Wambacq et al., 2024).

| Dog breed | Age | Gender | Dog size | BCS | IBW (Kg) | FBW (Kg) | Senior State | Treatment |
| --- | --- | --- | --- | --- | --- | --- | --- | --- |
| Witte herder | 9 | Female | Large | 4 | 23.7 | 24.5 | Geriatric | Control |
| Beagle | 7 | Male | Medium | 4 | 11.0 | 10.9 | Senior | Control |
| Mechelaar | 12 | Female | Large | 6 | 34.6 | 34.9 | Geriatric | Control |
| Tervuerense herder | 12 | Female | Medium | 5 | 20.2 | 19.2 | Geriatric | Control |
| Boerboel | 8 | Male | Giant | 6 | 51.4 | 50.9 | Geriatric | Control |
| Cavalier King Charles Spaniel | 9 | Female | Small | 5 | 7.0 | 6.6 | Senior | Control |
| Border collie | 11 | Female | Medium | 5 | 19.6 | 19.7 | Geriatric | Control |
| Chihuahua | 9 | Male | Small | 6 | 3.3 | 3.2 | Senior | Control |
| Roemeense straathond | 10 | Female | Medium | 4 | 20.4 | 19.7 | Senior | scFOS+ |
| Kruising Teckel - King Charles Spaniel | 8 | Male | Small | 5 | 9.5 | 9.5 | Senior | scFOS+ |
| Berner Sennen x labrador | 9 | Male | Large | 6 | 40.4 | 41.4 | Geriatric | scFOS+ |
| Ierse wolfshond | 5 | Male | Giant | 6 | 78.0 | 78.5 | Senior | scFOS+ |
| Beagle | 7 | Female | Medium | 4 | 9.4 | 9.1 | Senior | scFOS+ |
| Mechelaar | 9 | Female | Large | 6 | 32.1 | 33.1 | Geriatric | scFOS+ |
| Jack Russell terrier | 13 | Male | Small | 5 | 7.0 | 6.8 | Geriatric | scFOS+ |
| Schipperke | 10 | Male | Medium | 5 | 8.9 | 8.5 | Senior | scFOS+ |

**Supplementary Table 2**. Differential analysis of the overall of the relative abundance (D28+D77) by ALDEX2.

| ASV | Control (%) | scFOS+ (%) | P value |
| --- | --- | --- | --- |
| *Megamonas* spp | 2.06±5.3^b^ | 10.82±14.8^a^ | 0.011035 |
| Family Bacteroidaceae | 3.22±3.9^b^ | 7.02±8.7^a^ | 0.011381 |
| *Bacteroides plebeius* | 0.4±0.8^b^ | 0.8±1^a^ | 0.014046 |
| Clostridiales ASV | 0.18±0.3^b^ | 0.46±0.7^a^ | 0.016684 |
| Family Lachnospiraceae | 25.01±19.8^a^ | 16.73±13.7^b^ | 0.028665 |
| *Phascolarctobacterium* | 0.18±0.2^b^ | 0.69±0.8^a^ | 0.04383 |
| Family Succinivibrionaceae | 0.34±0.6 | 0.81±1.7 | 0.052734 |
| *Prevotella copri* | 1.57±3.6 | 3.95±8 | 0.065749 |
| *Fusobacterium* spp | 1.15±1.3 | 3.52±4.7 | 0.066088 |
| *Sutterella* spp | 0.76±1.6 | 0.74±0.6 | 0.077665 |
| *Streptococcus luteciae* | 0.66±2.3 | 10.11±23.5 | 0.09696 |
| *Catenibacterium* spp | 1.51±3.5 | 1.32±1.9 | 0.099771 |
| *Fusobacterium* spp | 1.15±1.3 | 3.52±4.7 | 0.10822 |
| *Dorea* spp | 4.46±3.9 | 2.38±3 | 0.129395 |
| *Bacteroides* spp | 2.57±3.5 | 5.26±7.6 | 0.17189 |
| Mogibacteriaceae ASV | 0.2±0.3 | 0.01±0 | 0.17953 |
| *Blautia* spp | 3.52±4.6 | 2.24±2.9 | 0.191868 |
| Family Peptostreptococcaceae | 0.82±0.9 | 0.21±0.3 | 0.198066 |

Superscripts refers to significant differences (P<0.05).

**Supplementary Figure 1**. Rarefaction curves of good coverage and observed otus.


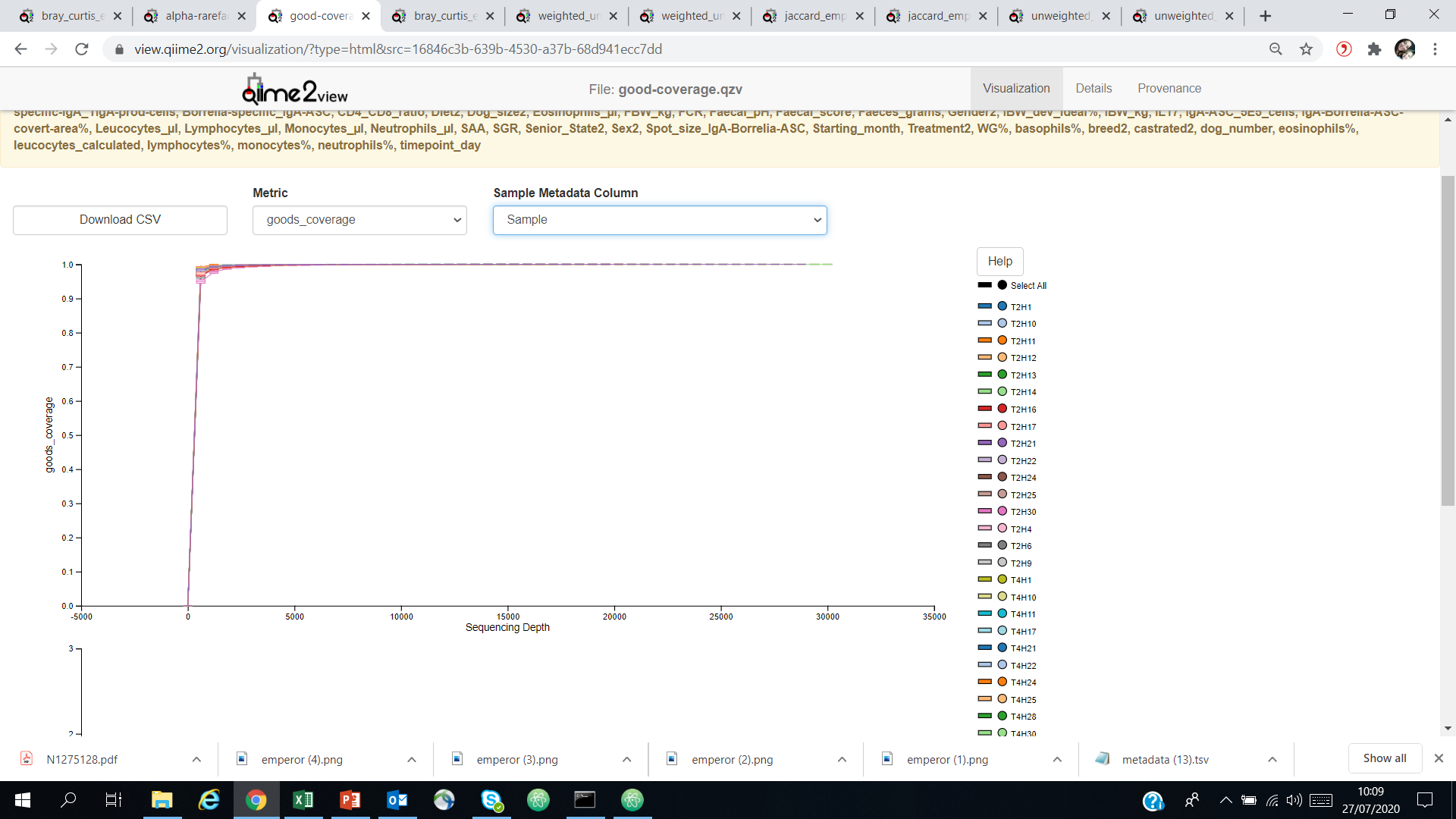

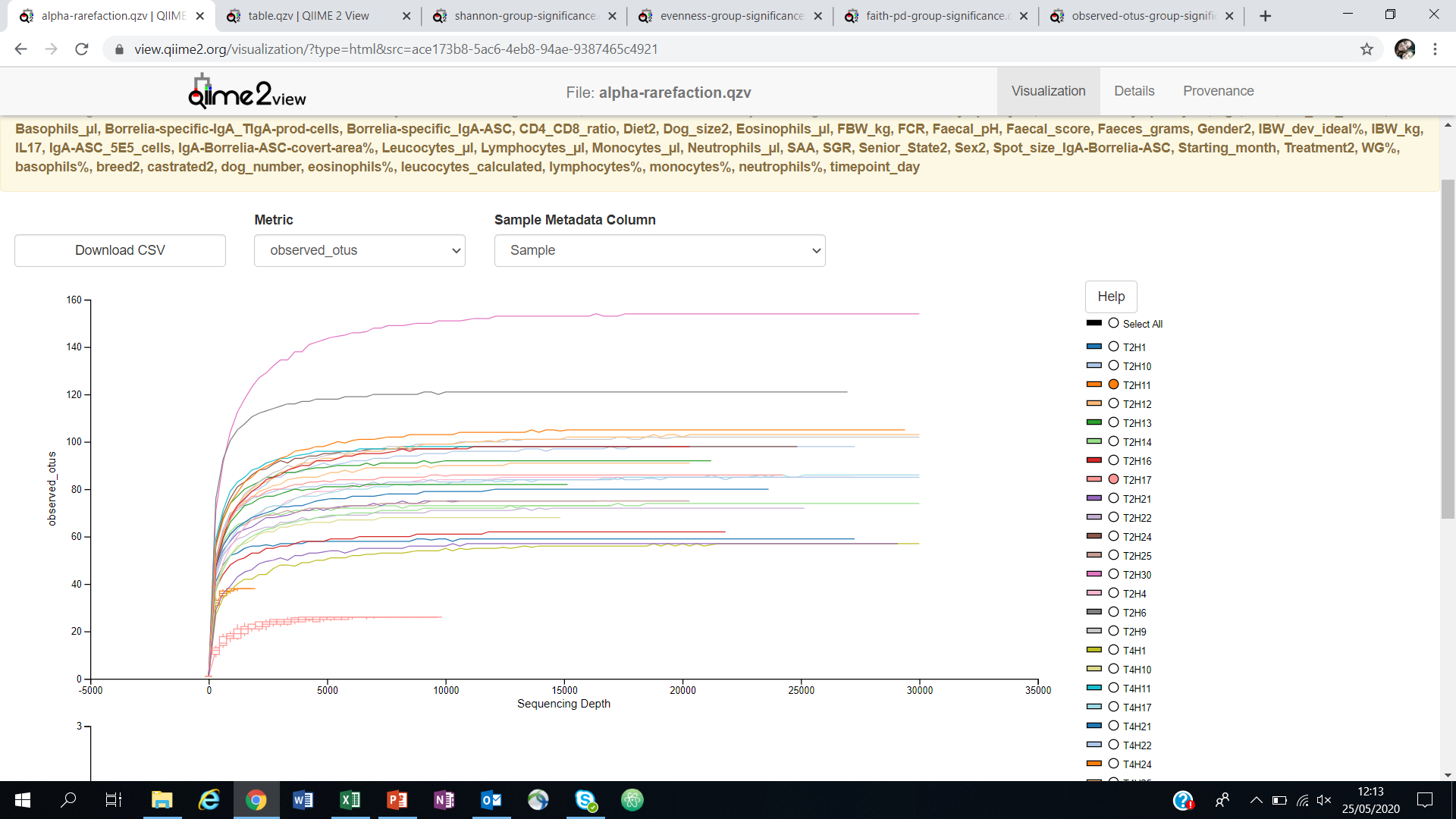


**Supplementary Figure 2**. Relative abundance of individual profiles at family level at day 28 and 77.


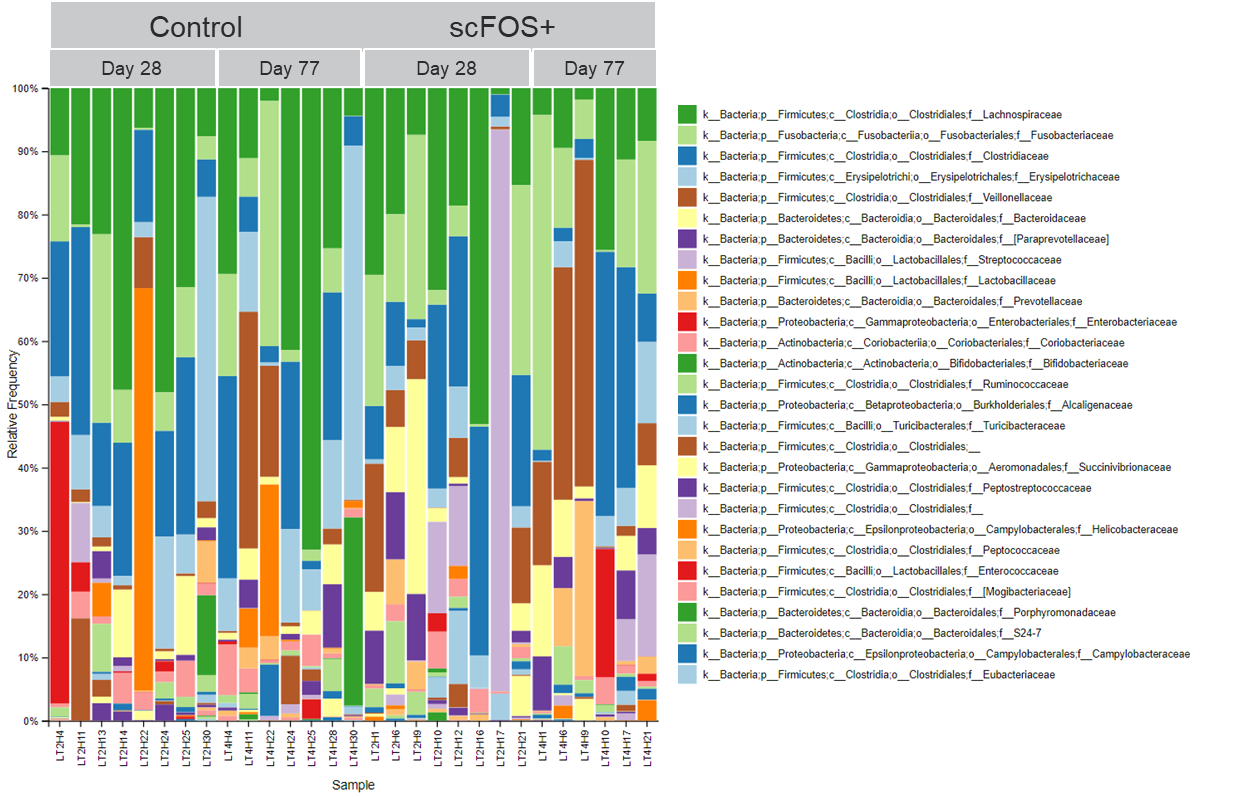

Supplement: Supplementary file 1 — Supplementary Material 1 [file 41598_2025_10280_MOESM1_ESM.docx]
